# An investigation of resistance to β-lactam antimicrobials among staphylococci isolated from pigs with exudative epidermitis

**DOI:** 10.1186/1746-6148-9-211

**Published:** 2013-10-17

**Authors:** Jeonghwa Park, Robert M Friendship, J Scott Weese, Zvonimir Poljak, Cate E Dewey

**Affiliations:** 1 Department of Population Medicine, Ontario Veterinary College, University of Guelph, 50 Stone Road, Guelph N1G2W1, ON, Canada; 2 Department of Pathobiology, Ontario Veterinary College, University of Guelph, 50 Stone Road, Guelph N1G 2W1, ON, Canada

**Keywords:** β-lactam resistance, Methicillin resistant *Staphylococcus aureus*, Methicillin-resistant *Staphylococcus hyicus*, Exudative epidermitis, Pigs, *mec*A gene, *mec*C (novel *mec*A_LGA251_), SCC*mec* type V

## Abstract

**Background:**

A high proportion of staphylococci isolated from pigs affected with exudative epidermitis were found to be resistant to β-lactam antimicrobials. The primary objective of this research was to investigate and characterize β-lactam resistance in *Staphylococcus hyicus*, *Staphylococcus aureus* and other staphylococci isolated from these pigs.

**Results:**

The antimicrobial resistance patterns of 240 staphylococci isolates were determined by disk diffusion, of which 176 (73.3%) of the isolates were resistant to 3 β-lactams (penicillin G, ampicillin, and ceftiofur). The presence of *mec*A gene was identified in 63 staphylococci isolates from skin samples by PCR. The *mec*A gene was identified in 19 *S*. *aureus*, 31 *S*. *hyicus*, 9 *Staphylococcus chromogenes*, 2 *Staphylococcus pseudintermedius* isolates, and in 1 isolate each of *Staphylococcus arlettae*, and *Staphylococcus cohnii* subspecies *urealyticus*. From SCC*mec* typing results, the majority (45/63, 71.4%) were shown to be SCC*mec* type V. One isolate was SCC*mec* III. Fourteen isolates were detected as *mec* class A, *mec* class C or *ccr* type 5. The *ccr* complex and *mec* complex was not detected in 3 isolates of methicillin resistant *S*. *hyicus* (MRSH) based on multiplex PCR. Of the 30 isolates of MRSA identified from nasal samples of the pigs, 29 isolates were SCC*mec* type V and 1 isolate was SCC*mec* type II. Staphyloccoci isolates that were *mec*A negative but resistant to β-lactam antimicrobials were further examined by screening for *mec*C, however all were negative. Furthermore, the majority of *mec*A negative β-lactam resistant staphylococci isolates were susceptible to oxacillin and amoxicillin-clavulanic acid in a double disk diffusion test.

**Conclusions:**

Methicillin resistance can be identified in a variety of staphylococcal species isolated from pigs. In this study there was a great deal of similarity in the SCC*mec* types between staphylococcal species, suggesting that resistance may be passed from one species of staphylococci to another species of staphylococci. While this has been reported for acquisition of methicillin-resistance from coagulase negative staphylococci to *S*. *aureus*, these data suggest that transmission to or from the porcine pathogen *S. hyicus* may also occur. The identification of methicillin resistance in a variety of staphylococcal species in pigs does raise concerns about the spread of serious multi-drug resistance in food producing animals and warrants further study.

## Background

Exudative epidermitis occurs as a generalized or localized skin disease of young pigs. The disease is common, can result in high morbidity and if severe can cause high death losses [1]. Resistance to β-lactam antimicrobials among staphylococci isolated from pigs is common [2-5], but few studies have been conducted to examine this topic, apart from relatively recent studies that have evaluated the prevalence of methicillin-resistant *Staphylococcus aureus* (MRSA) colonization in healthy pigs. Public health concerns associated with MRSA in pigs have generated a great deal of interest, and numerous studies have documented the widespread prevalence of MRSA among the world pig population [6-9]. However, *S*. *aureus* has only minor significance as a swine pathogen, whereas other staphylococci such as *Staphylococcus hyicus* can result in economically important disease such as exudative epidermitis [10,11]. Previous studies by our group have identified that treatment failure in cases of exudative epidermitis in pigs is common, partly due to the widespread presence of multi-drug resistance in staphylococci [5]. Resistance against the β-lactam family of antibiotics including penicillin G, ampicillin and cephalosporins was particularly noteworthy because penicillin is typically the drug of choice in the treatment of exudative epidermitis, almost exclusively in the absence of culture and susceptibility testing [5].

Acquired resistance to β-lactams is mediated through two main mechanisms, β-lactamase production or altered penicillin binding protein (PBP2a) production. Bacterial β-lactamases hydrolyze the β-lactam ring and in staphylococci typically confer resistance to penicillins (including amoxicillin and ampicillin). Inhibitors of β-lactamase (clavulanate, sulbactam, and tazobactam) can inhibit this resistance mechanism and β-lactam/β-lactamase inhibitor combinations are widely used in some species, but not in the swine industry [12]. In contrast, altered PBP2a production encoded by *mec*A, results in low affinity for all β-lactams and confers broad resistance to β-lactams (including cephalosporins and carbapenems) that is not affected by β-lactamase inhibitors.

Despite the importance of *S*. *hyicus* in swine disease, the commonness of β-lactam use to treat staphylococcal infections, and clinical evidence indicating poor response of exudative epidermitis to β-lactams in some situations [5], there has been minimal investigation of β-lactam antimicrobial resistance in this species. The primary objective of this research was to investigate and characterize β-lactam resistance in *S*. *hyicus*, *S*. *aureus* and other staphylococci isolated from pigs affected with exudative epidermitis.

## Methods

### Source of staphylococci isolates

Staphylococcal isolates were obtained from a previous trial [5]. Briefly, researchers visited 30 farms with endemic exudative epidermitis, taking skin and nasal swabs of pigs (6 pigs per farm on average) with clinical signs of exudative epidermitis. One hundred and forty-four *S*. *hyicus* and 96 *S*. *aureus* isolates from skin samples were identified by the Animal Health Laboratory (AHL), University of Guelph, Ontario with standard laboratory techniques including colony morphology, haemolysis, Gram stain, catalase reaction and coagulase reaction. Antimicrobial susceptibility testing for penicillin G (10 units), ampicillin (10μg) and ceftiofur (30μg) were performed by disk diffusion (Kirby-Bauer procedure) as per Clinical and Laboratory Standard Institution (CLSI) guidelines [13] by AHL. There were 71.5% (103/144) of *S*. *hyicus* isolates and 76% (73/96) of *S*. *aureus* isolates concurrently resistant to penicillin G, ampicillin and ceftiofur. Additionally, 30 MRSA isolates were recovered from nasal swabs. Nasal swabs were placed in enrichment broth and incubated for a day and then inoculated onto MRSA chromogenic agar (BBL CHROM agar MRSA, Becton Dickinson, SparksMD) and incubated aerobically for 24-48 h. Isolates were identified as *S*. *aureus* by Gram stain, catalase test, tube coagulase test and the *S*. *aureus* latex agglutination assay (Pastorex Staph plus, Bio Rad Laboratories Ltd, Mississauga, ON). Staphylococcal isolates that were resistant to penicillin G, ampicillin, or ceftiofur from skin samples and MRSA from nasal swabs were included in this study.

### Staphylococcus speciation

Methicillin-resistant staphylococci that were presumed to be *S*. *hyicus* based on standard phenotypic identification were confirmed by *S*. *hyicus* PCR [14], *sod*A sequencing, or matrix assisted laser desorption/ionization – time of flight (MALDI-TOF) MS analysis by a Micro flex LT mass spectrometer (Bruker Daltonik) using the MALDI Biotyper software package with the reference database v.2.0 by AHL.

### Methicillin resistance

Methicillin-resistance was evaluated by *mec*A PCR or detection of PBP2a by latex agglutination test (LAT) (Oxoid, Hants, UK). Amplification of *mecA* DNA with the primers 5’-GTT GTA GTT GTC GGG TTT GG-3’ and 5’CTT CCA CAT ACC ATC TTC TTT AAC-3’, using previously described conditions [15] was performed. Isolates that were *mecA*-negative but resistant to ceftiofur were also tested for the novel *mec* homologue *mecC* using a multiplex PCR [16]. The primers for *mecC* were 5’-GAA AAA AAG GCT TAG AAC GCC TC- 3’ and 5’-GAA GAT CTT TTC CGT TTT CAG C-3’. All amplifications were performed on supernatants from crude DNA extracts which were prepared and purified with InstaGene™ Matrix (Bio-Rad Laboratories, Hercules, Canada). Products of PCR were electrophoresed through 1.5% agarose gels and visualized through GelRed nucleic acid stain (BIOTIUM, Hayward, CA, USA).

### *spa* typing

Thirty-nine MRSA isolates of 49 total MRSA isolates were selected to type the staphylococcal protein A gene (*spa* typing) [17].

### SCC*mec* typing

All methicillin-resistant isolates were further characterized based on SCC*mec* elements using multiplex PCRs (M-PCRs) for typing of the *mec* complex class A and B, and the *ccr* complex type 1, type 2, and type 3 (M-PCR 1), for typing of *ccr* complex type 5 (M-PCR 2), for typing the *mec* complex class C (M-PCR 3), and for typing the *ccr* complex type 4 (M-PCR 4). The primer pairs used for M-PCR 1 and M-PCR 2 were referred from Zhang et al. [18] and the primer pairs used for M-PCR 3 and M-PCR 4 were referred from Kondo et al. [19].

### Effect of β-lactamase inhibitors

Staphylococcal skin isolates that were resistant to penicillin G, ampicillin and ceftiofur but *mecA* gene negative were tested for susceptibility to oxacillin and amoxicillin/clavulanic acid by disk diffusion [13]. Synergy between oxacillin and amoxicillin/clavulanic acid was detected by a double-disk diffusion test where a disk of amoxicillin/clavulanic acid (20ug/10ug, respectively) and a disk of oxacillin (1ug) were placed 15mm apart (center to center) on an inoculated agar plate. A clear extension of the edge of the oxacillin inhibition zone toward the disk containing clavulanic acid was interpreted as synergy, indicating the presence of a β-lactamase [20].

## Results

**Figure 1:**
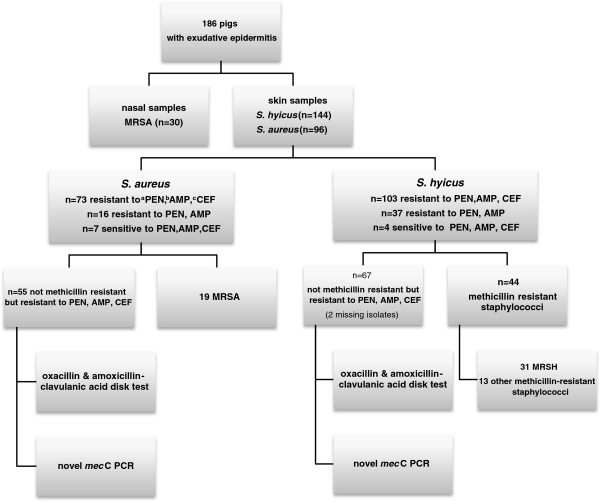
**A flowchart of the steps taken to examine the staphylococci isolates from pigs.**^a^PEN: penicillin G ^b^AMP: ampicillin ^c^CEF: ceftiofur.

A flow chart describing the testing and the results is presented in Figure 1. One hundred forty-four presumed *S*. *hyicus* and 96 *S*. *aureus* isolates from skin swabs were further tested. The *mec*A was identified in 19 *S*. *aureus* skin isolates and 44 presumed *S*. *hyicus* skin isolates. Thirty-one (70.5%) of the presumptive *S*. *hyicus* isolates were reconfirmed as *S*. *hyicus* (MRSH), 9 were methicillin-resistant *S*. *chromogenes* (MRSC), 2 were methicillin-resistant *S*. p*seudintermedius* (MRSP) and one each of *S*. *arlettae* and *S*. *cohnii* subsp. *urealyticus*. The thirty nasal isolates had been previously confirmed as MRSA. The characteristics of these methicillin-resistant staphylococci isolates are overviewed in Table 1.

**Table 1:** **Antimicrobial resistant characterization of methicillin**-**resistant staphylococci isolated from pigs with exudative epidermitis**

**Farms**	**Pigs**	**Species**	**Spa types**	**SCC *mec *** **type**	**Disk diffusion susceptibility test**		
					**Penicillin G**	**Ampicillin**	**Ceftiofur**
1	1	MRSA(N) ^a^	t034(539)	SCC*mec* typeV	NA	NA	NA
	2	MRSH^c^	NA	*mec* class C	R^h^	R	R
		MRSA(S) ^b^	t034(539)	SCC*mec* typeV	R	R	R
		MRSA(S)	t034(539)	SCC*mec* typeV	R	R	R
	3	MRSA(N)	t034(539)	SCC*mec* typeV	NA	NA	NA
		MRSC^d^	NA	*ccr* type 5	R	R	R
	4	MRSA(N)	t034(539)	SCC*mec* typeV	NA	NA	NA
2	1	MRSH	NA	SCC*mec* typeV	R	R	S^i^
	2	MRSH	NA	SCC*mec* typeV	R	R	R
3	1	MRSH	NA	SCC*mec* typeV	R	R	R
	2	MRSH	NA	SCC*mec* typeV	R	R	R
4	1	MRSA(N)	t034(539)	SCC*mec* typeV	NA	NA	NA
		MRSA(S)	t034(539)	SCC*mec* typeV	R	R	R
		MRSA(S)	t034(539)	SCC*mec* typeV	R	R	R
	2	MRSA(N)	t034(539)	SCC*mec* typeV	NA	NA	NA
		MRSA(S)	t034(539)	SCC*mec* typeV	R	R	R
		MRSA(S)	t034(539)	SCC*mec* typeV	R	R	R
		MRSAr^e^	NA	SCC*mec* typeV	R	R	R
	3	MRSA(N)	t034(539)	SCC*mec* typeV	NA	NA	NA
		MRSA(S)	t034(539)	SCC*mec* typeV	R	R	R
		MRSA(S)	t034(539)	SCC*mec* typeV	R	R	R
	4	MRSA(N)	t026 (93)	SCC*mec* typeV	NA	NA	NA
		MRSA(S)	t034(539)	SCC*mec* typeV	R	R	R
		MRSCo^f^	NA	SCC*mec* typeV	R	R	R
	5	MRSA(N)	t034(539)	SCC*mec* typeV	NA	NA	NA
		MRSA(S)	t034(539)	SCC*mec* typeV	R	R	R
	6	MRSA(N)	t034(539)	SCC*mec* typeV	NA	NA	NA
		MRSA(S)	t034(539)	SCC*mec* typeV	R	R	R
		MRSA(S)	t034(539)	SCC*mec* typeV	R	R	R
5	1	MRSH	NA	*ccr* type 5	R	R	R
6	1	MRSH	NA	SCC*mec* typeV	R	R	R
	2	MRSH	NA	untypeable	R	R	R
7	1	MRSH	NA	*mec* class A	R	R	R
	2	MRSH	NA	*mec* class A	R	R	R
	3	MRSH	NA	*mec* class A	R	R	R
		MRSH	NA	untypeable	R	R	R
	4	MRSH	NA	*mec* class A	R	R	R
		MRSH	NA	untypeable	R	R	R
	5	MRSH	NA	*mec* class C	R	R	R
8	1	MRSH	NA	SCC*mec* typeV	R	R	S
9	1	MRSH	NA	SCC*mec* typeV	R	R	R
	2	MRSH	NA	*ccr* type 5	R	R	R
10	1	MRSA(N)	t034(539)	SCC*mec* typeV	NA	NA	NA
	2	MRSH	NA	SCC*mec* typeV	R	R	R
11	1	MRSA(N)	t8588	SCC*mec* typeV	NA	NA	NA
	2	MRSA(N)	t034(539)	SCC*mec* typeV	NA	NA	NA
	3	MRSA(N)	t034(539)	SCC*mec* typeV	NA	NA	NA
	4	MRSA(N)	t011	SCC*mec* typeV	NA	NA	NA
12	1	MRSH	NA	SCC*mec* typeV	R	R	R
	2	MRSP^g^	NA	SCC*mec* typeV	R	R	R
13	1	MRSA(S)	t034(539)	SCC*mec* typeV	R	R	R
	2	MRSP	NA	*ccr* type 5	R	R	R
		MRSC	NA	*ccr* type 5	R	R	R
	3	MRSC	NA	SCC*mec* typeIII	R	R	R
	4	MRSC	NA	*ccr* type 5	R	R	R
		MRSH	NA	*ccr* type 5	R	R	R
14	1	MRSH	NA	SCC*mec* typeV	R	R	R
15	1	MRSA(N)	t002(2)	SCC*mec* typeII	NA	NA	NA
	2	MRSC	NA	SCC*mec* typeV	R	R	R
16	1	MRSA(S)	t034(539)	SCC*mec* typeV	S	S	S
17	1	MRSH	NA	SCC*mec* typeV	R	R	S
	2	MRSH	NA	SCC*mec* typeV	R	R	S
	3	MRSH	NA	SCC*mec* typeV	R	R	R
18	1	MRSA(N)	t034(539)	SCC*mec* typeV	NA	NA	NA
	2	MRSA(N)	t034(539)	SCC*mec* typeV	NA	NA	NA
		MRSA(S)	NA	SCC*mec* typeV	R	R	R
	3	MRSA(N)	t034(539)	SCC*mec* typeV	NA	NA	NA
		MRSA(S)	NA	SCC*mec* typeV	R	R	R
	4	MRSA(N)	t034(539)	SCC*mec* typeV	NA	NA	NA
		MRSA(S)	NA	SCC*mec* typeV	R	R	R
		MRSH	NA	SCC*mec* typeV	R	R	R
	5	MRSA(N)	t034(539)	SCC*mec* typeV	NA	NA	NA
		MRSA(S)	NA	SCC*mec* typeV	R	R	R
	6	MRSA(N)	t034(539)	SCC*mec* typeV	NA	NA	NA
19	1	MRSA(N)	t034(539)	SCC*mec* typeV	NA	NA	NA
		MRSC	NA	SCC*mec* typeV	R	R	R
		MRSC	NA	SCC*mec* typeV	R	R	R
	2	MRSA(N)	t034(539)	SCC*mec* typeV	NA	NA	NA
		MRSC	NA	SCC*mec* typeV	R	R	R
		MRSC	NA	*ccr* type 5	R	R	R
	3	MRSA(N)	t034(539)	SCC*mec* typeV	NA	NA	NA
	4	MRSA(N)	t034(539)	SCC*mec* typeV	NA	NA	NA
	5	MRSA(N)	t1298	SCC*mec* typeV	NA	NA	NA
	6	MRSA(N)	t034(539)	SCC*mec* typeV	NA	NA	NA
		MRSA(S)	t571(109)	SCC*mec* typeV	R	R	S
20	1	MRSA(N)	t034(539)	SCC*mec* typeV	NA	NA	NA
	2	MRSA(N)	t034(539)	SCC*mec* typeV	NA	NA	NA
		MRSH	NA	SCC*mec* typeV	R	R	S
	3	MRSH	NA	SCC*mec* typeV	R	R	S
		MRSH	NA	SCC*mec* typeV	R	R	R
	4	MRSA(N)	t034(539)	SCC*mec* typeV	NA	NA	NA
	5	MRSH	NA	SCC*mec* typeV	R	R	S
	6	MRSH	NA	SCC*mec* typeV	R	R	S

^a^MRSA(N): methicillin-resistant *Staphylococcus aureus* recovered from nasal swabs.

^b^MRSA(S): methicillin-resistant *Staphylococcus aureus* recovered from skin swabs.

^c^MRSH: methicillin-resistant *Staphylococcus hyicus* recovered from skin swabs.

^d^MRSC: methicillin-resistant *Staphylococcus chromogenes* recoverd from skin sw.abs

^e^MRSAr: methicillin-resistant *Staphylococcus arlettae* recovered from skin swabs.

^f^MRSCo: methicillin-resistant *Staphylococcus cohnii* recovered from skin swabs.

^g^MRSP: methicillin-resistant *Staphylococcus pseudintermidius* recovered from skin swabs.

^h^R: resistant.

^i^S: susceptible.

NA: not applicable.

The overall prevalence of methicillin resistance on farms was found to be 50% (15/30), 20% (6/30), and 26.7% (8/30) for MRSH, MRSA (skin samples), and MRSA (nasal samples), respectively. Twenty farms out of 30 farms had methicillin-resistant staphylococci from either the skin or nasal samples. Five farms had pigs harbouring both MRSA (skin and nose samples) and MRSH, and 5 farms had pigs harbouring both MRSA (skin and nasal samples) and methicillin- resistant non- *S*. *aureus* staphylococci (excluding MRSH). Seven spa types were detected from 39 MRSA isolates. The spa type t034 (539), a common ST398 strain, was predominant (84.6%). The ST398-associated t571 and t011 were found in single pigs and *spa* types t002, t026, t8588 and t1298 were also found once each.

The majority of SCC*mec* types in methicillin-resistant staphylococci in the study were SCC*mec* type V (75.3%) (Table 2), with SCC*mec* type V accounting for 97.6% of MRSA (48/49) and 61.3% of MRSH (19/31) isolates. Seventeen isolates only yielded results for *mec* gene complex class A or class C, or *ccr* gene complex type 5. Three MRSH had any neither *mec* gene complex nor *ccr* gene complex, and so SCC*mec* typing was repeated for these non- typeable isolates. Multiple isolates with the same incomplete SCC*mec* typing results were found on 2 farms. While 2 different staphylococcal species possessing the same complete SCC*mec* type were found on 5 farms. Three pigs were found to harbour both MRSH and MRSA (nasal samples) in the same SCC*mec* type V. One pig had harboured both MRSH and MRSA (skin), both of which harboured *mec* gene complex class C. Four other pigs harboured both MRSA and other methicillin-resistant staphylococci (other than MRSH), and they possessed SCC*mec* typeV or *ccr* gene complex type 5.

**Table 2:** **Overview of SCC *mec *elements found 93 methicillin**-**resistant staphylococci isolated from pigs with exudative epidermitis**

***ccr-*** **complex**	***mec-*** **complex**	**SCC *mec *** **type**	**n. isolates**	**Species(n.isolates)**
*ccr* type 2	class A	II	1	*S*. *aureus *(1)
*ccr* type 3	class A	III	1	*S*. *chromogenes *(1)
*ccr* type 5	class C	V	74	*S*. *hyicus *(19), *S*. *aureus* (48), *S*. *chromogenes* (4), *S*. *pseudintermedius *(1), *S*. *arlettae* (1), *S*. *cohnii* subsp. *Urealyticus *(1)
*Ccr* type 5	NF	NT type3^b^	8	*S*. *hyicus *(3), S. chromogenes (4), *S*. *pseudintermedius *(1)
NF^a^	class A	NT type2	4	*S*. *hyicus *(4)
NF	class C	NT type 1	2	*S*. *hyicus *(2)
NF	NF	NT type 4	3	*S*. *hyicus *(3)

^a^NF: non finder.

^b^NT: non-typeable cassette; the categorization into ‘type 1,2,3, and 4’ was done on the moment of detection.

Sixty-seven isolates of *S*. *hyicus* were *mecA* negative but resistant to 3 β-lactam antimicrobials (penicillin G, ampicillin and ceftiofur). Sixty-two (92.5%) were susceptible to both oxacillin and amoxicillin-clavulanic acid, while 3 (4.5%) were resistant to oxacillin but susceptible to amoxicillin-clavulanic acid and 2 (3.0%) were susceptible to oxacillin but resistant to amoxicillin-clavulanic acid. Further, the 3 oxacillin-resistant, amoxicillin-clavulanic acid susceptible isolates did not show an alteration of the zone of inhibition in the double disk test. These 67 *S*. *hyicus* isolates tested negative for *mecC*.

Fifty-five isolates of *S*. *aureus* were *mecA* negative but resistant to the penicillin G, ampicillin and ceftiofur. Twenty-six (47.3%) of these were susceptible to both oxacillin and amoxicillin-clavulanic acid and 8 (14.5%) isolates were resistant to oxacillin but susceptible to amoxicillin-clavulanic. Twenty-one isolates (38.2%) were resistant to both oxacillin and amoxicillin-clavulanic acid but, when the double disk test was used, alteration of the zone of inhibition was apparent for 20 (95.2%) isolates; suggesting oxacillin resistance was mediated through excessive β-lactamase production. These 55 *S*. *aureus* isolates were *mecC* negative.

## Discussion

There has been considerable work done to examine the prevalence of MRSA in the pig population [6-9] and its association with human infection because of the potential public health risk [21]. MRSA is presumed to have acquired *mecA* from coagulase negative staphylococci [22], and methicillin-resistant coagulase negative staphylococci are common commensals [14,22]. Methicillin-resistance has emerged, presumably in a similar manner, in other pathogenic staphylococci. Therefore, it is reasonable to consider that transmission of *mecA* between *S*. *aureus* and *S*. *hyicus*, in either direction, might occur on pig farms where both staphylococcal species are present, particularly in the presence of regular therapeutic or prophylactic use of β-lactam antimicrobials.

The finding of *mecA* in *S*. *hyicus* is important for a number of reasons. From a swine health standpoint, it is of relevance because of resistance to penicillin and ceftiofur, two commonly used antimicrobials [23]. From a public health standpoint the finding that MRSA and MRSH can carry the same SCC*mec* raises the concern that the genetic material conferring multiple antimicrobial resistance may be passing from species to species within the bacterial population of a farm, and raises questions about whether MRSH could ultimately be a source for further emergence of MRSA clones on pig farms. Finding the same SCC*mec* does not indicate whether *mecA* was transmitted from *S*. *aureus* to *S*. *hyicus*, from *S*. *hyicus* to *S*. *aureus* or to both *S*. *hyicus* and *S*. *aureus* from another source. Further study of transmission of *mecA* between different staphylococci at the pig or farm level is warranted. Thus, while *S*. *hyicus* is of little zoonotic relevance, the public health relevance of MRSH cannot be completely dismissed.

SCC*mec* type was not determined for 38.7% of MRSH, 44.4% of MRSC, and 50% of MRSP because the primers that were used were unable to detect either the *mec* or *ccr* complexes (n=3), only detecting the *mec* complex (n=6) or only detecting the *ccr* complex (n=8). This is not unexpected since there has been limited investigation other than in MRSA. SCC*mec* types other than those evaluated here are described and it is possible that further testing would have clarified the SCC*mec* type in the isolates in the present study. However, even with testing for all described SCC*mec* types, some isolates may remain untypable [24] and it was determined that additional typing would have contributed little to fulfilling the main objectives of this study.

One curious finding here was the presence of isolates that were resistant to β-lactams and β-lactam/ β-lactam inhibitor combinations but *mecA* negative. To the authors’ knowledge, the only known mechanism for ceftiofur and amoxicillin-clavulanic acid resistance in staphylococci is methicillin resistance, and this suggests that a different *mec* element that is not detectable by conventional *mecA* PCR or PBP2a LAT could be involved. One recent example of this is *mecC* (previously referred to as *mecA*_LGA251_) which has been identified in animals and humans in Europe [16,20,25]. However, this mecC gene was not detected. This does not exclude the possibility that a different novel *mec* element was present. It is noteworthy that double disk diffusion testing indicated an impact of clavulanic acid on oxacillin resistance, suggesting that hyperproduction of β-lactamase might be the cause of the resistance for some (n=20) *mec*A negative β-lactam resistant *S*. *aureus* isolates.

## Conclusions

Methicillin resistance can be identified in a variety of staphylococcal species isolated from pigs. In this study there was a great deal of similarity in the SCC*mec* types between staphylococcal species, suggesting that resistance may be passed from one species of staphylococci to another species of staphylococci. While this has been reported for acquisition of methicillin-resistant from coagulase negative staphylococci to *S*. *aureus*, these data suggest that transmission to or from the porcine pathogen *S. hyicus* may also occur. The identification of methicillin resistance in a variety of staphylococcal species in pigs does raise concerns about the spread of serious multi-drug resistance in food producing animals and warrants further study.

## Competing interests

The authors acknowledge no conflict of interest.

## Authors’ contributions

All authors contributed to the writing of the paper. JP was primarily responsible for collecting the samples and performing the laboratory tests. All authors read and approved the manuscript. 
